# The diagnostic yield of molecular karyotyping: a retrospective single-center study

**DOI:** 10.3325/cmj.2025.66.92

**Published:** 2025-04

**Authors:** Emine Göktaş, Ahmet Burak Arslan, Betül Turan, Betül Okur Altındaş, Ayşe Gül Zamani, Mahmut Selman Yıldırım

**Affiliations:** 1 Department of Medical Genetics, Faculty of Medicine, Necmettin Erbakan University, Konya, Turkey; 2 Department of Medical Genetics, Kocaeli City Hospital, Kocaeli, Turkey; 3 Department of Medical Genetics, Samsun Training and Research Hospital, Samsun, Turkey

## Abstract

**Aim:**

To determine the diagnostic yield of chromosomal microarray analysis (CMA) in different patient groups: intellectual disability and developmental delay (ID/DD), multiple congenital anomalies (MCA), epilepsy, autism spectrum disorder (ASD), reproductive abnormalities, and dysmorphic features.

**Methods:**

We retrospectively reviewed microarray data of 176 patients admitted to the Medical Genetics Outpatient Clinic of Necmettin Erbakan University Medical Faculty Hospital from 2016 to 2022. After the copy number variation (CNV) interpretation, we evaluated the diagnostic strength of CMA in each group.

**Results:**

Phenotype-associated CNVs were detected in 20.3% (22/108) of patients with ID/DD, 23.9% (17/71) of patients with MCA, 15.9% of patients (7/44) with epilepsy, 16.6% (4/24) of patients with ASD, and 11.7% (2/17) of those with reproductive abnormalities. Chromosomal gains or losses were found in 43% (35/80) of patients with dysmorphic findings.

**Conclusion:**

This study confirmed the remarkable diagnostic yield of CMA in ID/DD, MCA, and ASD patients, and expanded its value for cases with epilepsy and dysmorphism.

The human genome is an ever-changing mechanism with diverse genomic alterations. Any deviation within a given genomic coordinate from one reference nucleotide to another is described as a single-nucleotide variation. The gene in question remains diploid, but its nature has been altered. Sometimes, not the type but only the “quantity” of a given position differs. Variations of this kind greater than 50 base pairs are collectively defined as copy number changes (CNC). CNC are classified into copy number variations (CNV) (germline) and copy number abnormalities (CNA) (somatic) (1).

Chromosomal microarray analysis (CMA) reveals chromosomal imbalances that are too small to be detected by a conventional G-banded karyogram; either by array comparative genomic hybridization (CGH) or by a single nucleotide polymorphism (SNP) array. The procedure allows measurement of changes in DNA sequence copy number along with simultaneous mapping of the defined sites to the genomic sequences. Hybridization with a great number of probes (called reporter sequences), representing the entirety of the genome, increases the precision of the technique within a single experiment (2,3). Unlike standard karyotyping, CMA is able to confirm microdeletions and microduplications as small as a few kilobases (4). It can be used in all groups of patients and is especially effective in a particular subset of complaints. CMA is recommended by the International Standard for Cytogenomic Arrays Consortium as a first-tier diagnostic tool to identify the underlying molecular mechanism of developmental delay/intellectual disability (ID/DD), multiple congenital anomalies (MCA), and autism spectrum disorders (ASD), with a diagnostic yield of 15%-20% (5). In epilepsy patients, the diagnostic yield was 8%-17% (6,7).

The phenotypic effects of CNVs lie along a continuous spectrum: from adaptive traits to embryonic lethality, and clinical genetic analysis is used to discriminate between pathogenic variants and benign ones (8). With underlying factors, such as phenotypic heterogeneity, that impede genotype-phenotype correlation attempts, current literature data are limited, and disease-associated CNV segments are exceedingly few. DECIPHER, a curated database, currently defines 66 pathogenic CNVs. A recent comprehensive meta-analysis with data from 753 994 individuals lists 128 disease-associated CNVs (9). In this context, the major challenge is the growing number of variants of unknown significance (VUS), which are suspected to be involved in disease but for which additional population-level data are required. This study evaluates the utility of CMA across a wide range of phenotypes. We aimed to compare its diagnostic yield among distinct patient subgroups to help prioritize those who may benefit most from this testing and to deepen our understanding of the nature and clinical relevance of genomic copy number imbalances.

## Patients and methods

The study enrolled 176 patients referred to the Medical Genetics Outpatient Clinic of Necmettin Erbakan University Medical Faculty Hospital from 2016 to 2022 for microarray analysis, with no prior selection criteria applied. The patients underwent medical history taking, physical examination, anthropometric measurements, at least three-generation family pedigree analysis, and relevant radiographic and biochemical tests. Those without apparent genetic etiology underwent microarray testing. Peripheral blood samples were obtained, and genomic DNA was extracted with MagNA Pure Compact Nucleic Acid Isolation Kit I (Roche Diagnostics, Rotkreuz, Switzerland). The analysis was performed with the Affymetrix Cytoscan Optima Array kit (Thermo Fisher Scientific, Waltham, MA, USA), and raw data were analyzed with Chromosome Analysis Suite (ChAS version 3.1.1.27, Thermo Fisher Scientific) in 131 patients. The detection rate for genomic losses was 200 kilobases, and for genomic gains it was 500 kilobases. The results of the remaining 45 patients were analyzed with the HumanCytoSNP-12 v.2.1 platform and BlueFuse Multi 4.5 (32178) software system (both from Illumina Inc., San Diego, CA, USA), with a resolution of 100 kilobases for both genomic losses and gains. Each CNV was analyzed when more than six consecutive probes were involved in a segment. The selection was determined according to the laboratory’s available resources. After initial bioinformatics analysis, the detected CNVs were interpreted in terms of genotype-phenotype correlation and pathogenicity, using various databases (DGV, DECIPHER, OMIM, UniProt, Ensembl, USCS, ClinGen, and PubMed) including our in-house data set (1,9-14). The findings were described according to the International System for Human Cytogenomic Nomenclature (ISCN) (15) and classified according to the American College of Medical Genetics (16) standards and guidelines for the interpretation and reporting of postnatal constitutional CNVs, into five groups: pathogenic, VUS/likely pathogenic, VUS, VUS/likely benign, and benign.

A G-banded karyotype was obtained from a peripheral blood sample of 160 patients, imaged with Lucia Cytogenetics 1.5.6 Karyo (Laboratory Imaging s.r.o., Prague, Czechia), and analyzed. The results were described according to the latest ISCN standards. All molecular and clinical findings were studied and reviewed by the same medical geneticist physician.

## RESULTS

Out of 176 patients (mean age 12.1 years), 97 were female (55%). Patients were classified into five groups based on their reason for referral, as follows: 1) ID/DD, 2) ASD, 3) MCA, 4) epilepsy, and 5) reproductive abnormalities. Patients with major congenital anomalies in two or more major organ systems were defined as those with MCA (17).

**Figure 1:**
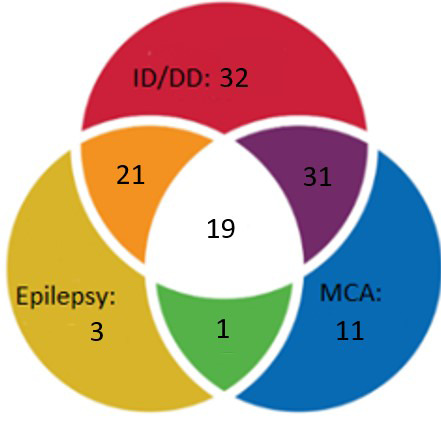
The overlaps between the major patient groups. IDD – Iintellectual disability and developmental delay; MCA – multiple congenital anomalies.

Only 32% (n = 56) of the patients were classified into isolated ID/DD, MCA, epilepsy, ASD, or reproductive abnormalities patient groups, as most of the patients had more than one main finding. The ID/DD group comprised 108 patients, MCA 71, epilepsy 44, ASD 24, and reproductive abnormalities 17. The most common overlap was between the groups with ID and MCA, with 50 patients showing both findings. Simultaneously, 40 patients had both epilepsy and ID, and 20 patients had epilepsy and MCA. In addition, 19 patients showed all three symptoms (Figure 1).

In terms of pathogenicity, the most common variants were VUS (28 variants in 26 patients), followed by pathogenic (24 variants in 18 patients) and likely pathogenic variants (18 variants in 15 patients). Table 1 shows the distribution of variant pathogenicity according to the patients’ clinical features. Normal microarray results and/or benign variants were identified in 117 patients.

**Table 1:** The pathogenicity of copy number variants (CNV) in different clinical groups

Patients	Pathogenic CNV	Likely pathogenic CNV	Variant of unknown significance CNV	Benign CNV/normal	Total
Intellectual disability and developmental delay	10	12	15	71	108
Epilepsy	4	3	5	32	44
Autism spectrum disorder	1	3	0	20	24
Multiple congenital anomalies	9	8	7	47	71
Reproductive anomalies	1	1	4	11	17

**Figure 2:**
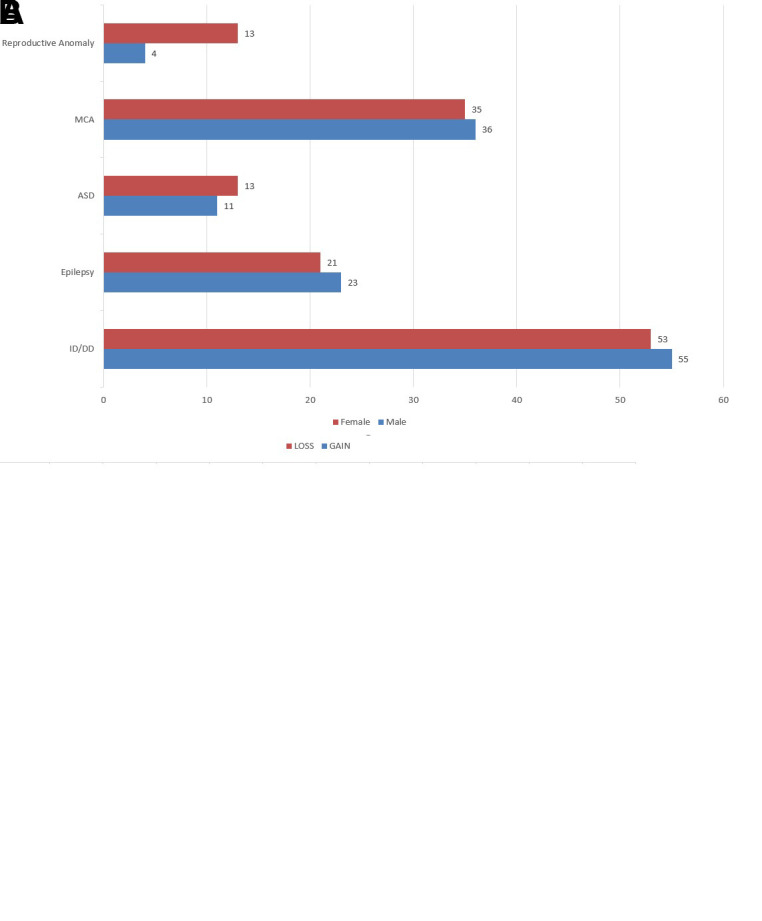
(**A**) Copy number gains or losses (**B**) Sex distibution across patient groups. IDD – intellectual disability and developmental delay; MCA – multiple congenital anomalies. ASD – autism spectrum disorder.

In the five clinical patient groups, the percentage of detected copy number gains and losses was mostly similar (Figure 2A). A considerable difference was observed only in the ASD group (4 copy number gains vs 1 loss). Sex distribution was also balanced overall (Figure 2B), but there was a female predominance in the reproductive abnormality group (13 vs 4).

**Figure 3:**
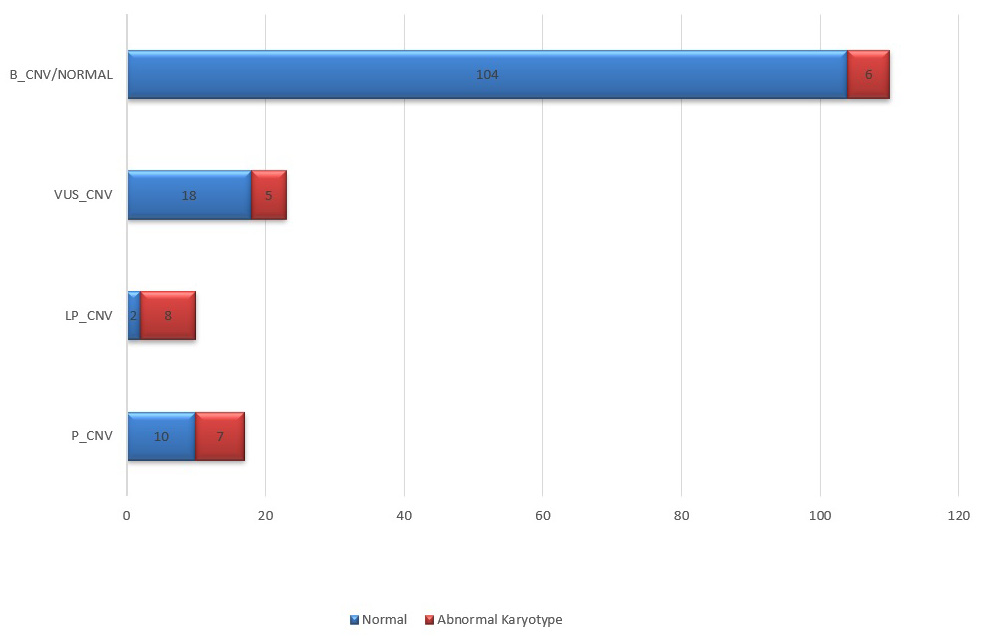
The number of patients with available conventional cytogenetics analysis results according to pathogenicity classification. CNV – copy number variation; VUS – variant of uncertain significance; LP – likely pathogenic; P – pathogenic.

Karyotype analysis was possible in 160 patients (Figure 3). An abnormal karyotype result was present in 7/17 patients in the pathogenic group (41%); 8/11 (72%) patients in the likely pathogenic group; and 5/20 (25%) patients in the VUS group, which made the additional diagnostic value of CMA 59%, 28%, and 75%, respectively.

The diagnostic yield was summarized in three classes: patients with at least one CNV classified as VUS, LP, or P; patients with at least one CNV classified as LP or P; and patients with at least one CNV classified as P. The detection rates in the three groups were 33% (n = 59), 18% (n = 33), 10% (n = 18), respectively. Diagnostic yields across clinical subgroups are summarized in Table 2.

**Table 2:** The detection rate of copy number variants (CNV) across different clinical phenotypes

		No. (%) patients with ≥1 CNV classified as		
Clinical phenotype	Total number of patients	VUS, LP, or P	LP or P	P
Intellectual disability and developmental delay	108	37 (34.2)	22 (20.3)	10 (9.2)
Multiple congenital anomalies	71	24 (33.8)	17 (23.9)	9 (12.6)
Epilepsy	44	12 (27.2)	7 (15.9)	4 (9)
Autism spectrum disorder	24	4 (16.6)	4 (16.6)	1 (4.1)
Reproductive anomalies	17	6 (35.2)	2 (11.7)	1 (5.8)
Dysmorphism	80	35 (43)	22 (27.5)	9 (11)

*Abbreviations: VUS – variant of uncertain significance; LP – likely pathogenic; P – pathogenic.

A segregation analysis by inheritance studies was possible for 13 CNVs in 13 patients (Table 3). Of those, 9 were *de novo* and 4 were parentally transmitted. All of the pathogenic CNVs were *de novo*. One of the *de novo* changes was the result of a parental reciprocal translocation between chromosomes 10 and 22. Among likely pathogenic CNVs, 4 were *de novo* and 1 was parentally transmitted. Among 5 VUS CNVs, 2 were *de novo* and 3 were parentally transmitted.

**Table 3:** The number of patients with available inheritance studies according to their variant classes

Classification	Inheritance studies	Total CNV*	*De novo*	Parental
Pathogenic CNV	3	3	3	-
Likely pathogenic CNV	5	5	4	1
Variant of unknown significance	5	5	2	3
Total	13	13	9	4

*CNV – copy number variation.

## DISCUSSION

In this study, we detected phenotype-associated CNVs in 20.3% (22/108) of patients with ID/DD, 23.9% (17/71) of patients with MCA, 15.9% of patients (7/44) with epilepsy, 16.6% (4/24) of patients with ASD, and 11.7% (2/17) of those with reproductive abnormalities. The primary focus of this study was the analysis of outcomes across different subsets of patients. This approach allows for a quantitative comparison of different outcomes across patient subgroups, providing an objective basis for identifying significant variations.

ID/DD subset includes an extremely broad spectrum of diseases (18). Many neurologic and metabolic diseases manifest with variable degrees of ID/DD, which complicates genotype-phenotype correlation attempts. The extensive application of array comparative genomic hybridization technology in recent years has enhanced the diagnostic efficacy for ID/DD cases. In a review of 33 studies comprising 21 698 patients, the diagnostic rate of the array in patients with ID/DD was between 11% and 15% (5). Another study found this rate to be 17.1% (19). In the current study, the ID/DD patient group had the highest rate of detected variants (P, LP, or VUS) among all the subgroups (34.2%). This finding highlights the relatively high frequency of VUS variants in this population. Furthermore, a notable proportion of patients with ID/DD appeared to benefit more directly from CMA studies, as 9.2% harbored P variants, and 20.3% had either P or LP variants.

MCA are commonly investigated in CMA studies because CNCs can result in different abnormalities in seemingly unrelated systems, in accordance with the function and the nature of the individual genes involved (20). This advantage showed itself in the highest diagnostic yields in this group. Among patients with MCA, LP or P variants were identified in 17 individuals (23.9%) and P variants alone were detected in 9 patients (12.6%). Furthermore, the overlaps between the three most common patient groups highlight the fact that CNCs, which involve multiple genes, affect multiple systems. Similarly, among 1227 Turkish patients with ID/DD, MCA, and autism spectrum disorders, 11% exhibited phenotype-associated CNVs (21).

Another important group examined in this study consisted of epilepsy patients. The International Standards for Cytogenomic Arrays Consortium does not list epilepsy as a phenotype for which CMA is a first-tier diagnostic tool. This is because many different sequence variants are also associated with different epilepsy syndromes, such as non-acquired focal epilepsy and well-defined generalized epilepsy (22). Baer et al reported the presence of pathogenic CNVs in 14% of 250 epilepsy patients. As a result, the authors recommended the use of CMA as a second-line diagnostic technique (23). In our study, pathogenic variants alone were identified in 9% of patients, while the combined detection rate of P and LP variants increased to 15.9%. When VUS was added, the yield reached as high as 27.2%, which shows the necessity to consider CMA in patients with epilepsy.

ASD has been the main focus of CMA studies to date (24). Yet, the complex nature of the disease and many underlying factors do not always allow a straightforward interpretation. Simons Foundation Autism Research Initiative Gene (25), a database dedicated to curating CNVs in the genes implicated in autism susceptibility, currently lists only 17 ASD-associated pathogenic CNV loci. In a diverse cohort of children with ASD, the diagnostic efficacy of CMA was 9%, while that of WES was 8% (26). In another study with a comparable design, the respective rates were 9.3% and 8.4% (27). In our cohort, 4 out of 5 changes in ASD patients were classified as VUS, which indicates the need for further studies. Pathogenic variants were observed in 4.1% of the patients. The detection rate of pathogenic CNVs was higher in epilepsy patients than in the ASD group (9% vs 4.1%), which further emphasizes the value of CMA in epilepsy.

CNVs in the genes controlling biological processes such as anatomical and functional development of reproductive organs, gametogenesis, and hormonal signaling might result in fertility-associated disorders (28,29). The great majority of CNVs within the reproductive anomaly group were only interpreted as VUS (66.6%). This was an expected outcome since CMA studies for these patients are scarcer than those for other patient groups. Further curation efforts to classify VUS variants are needed.

The detection rate of pathogenic CNVs in isolated neurodevelopmental disorders (NDD) was extremely lower than in patients with NDD-associated dysmorphic features, which indicates the predictive significance of dysmorphism to determine the molecular etiology (30). In the current study, patients with dysmorphic features had a notable proportion of pathogenic CNVs (11%). They also had the highest overall variant detection rate among all subgroups (VUS, LP, *P* = 43%). These findings highlight the importance of performing CMA analysis in dysmorphic patients.

In terms of gains and losses, interesting data were obtained. In patients with CNVs classified as VUS, LP, or P, the loss rate was 51% (36/70). On the other hand, in patients with CNVs classified as LP or P and in those with only P variants, the loss rates increased to 57% (24/42) and 58% (14/24), respectively. It seems that the loss of certain genes can be more easily associated with disease phenotypes. This is unsurprising since the loss of gene functions is better studied across the literature (31). In addition, copy number gains predominated among the variants only in the ASD subset (80%).

A significant proportion of all CNVs (62.5%) were *de novo* variants, with higher percentages observed in P and LP categories, which underscores the importance of segregation studies. Since the *de novo* CNVs occur at a rate of approximately 1.2% per genome per transmission and are more likely to explain the mechanism of a given phenotype, parentally transmitted variants need to be reconsidered in terms of their pathogenicity, taking into account expression variability (32). One out of the six well-defined pathogenic variants in our cohort was parentally transmitted, a finding indicating that parent analysis is crucial even for well-defined variants, since patients may not be aware of their clinical situation.

The detection rate of CNVs ranging from 16.6% to 43% in the phenotype groups is influenced by the criteria used for patient selection, the resolution of the array reagent kit, and the bioinformatic tools used. This study again demonstrated that microarray was not only an indispensable tool in the diagnostic evaluation of ID/DD, ASD, and MCA but also of epilepsy and dysmorphism. A large number of unsorted data in the literature causes many variants to stay within the VUS definition. The abundance of VUS variants shows the necessity for putative studies. A comprehensive analysis of these VUS patients and possible genotype-phenotype correlations will be further discussed in a separate study.

This study has several limitations. The small number of patients and a single-center setting limit the generalizability of the data. Besides, due to the retrospective design of the study, we evaluated the results obtained by two microarray platforms with different resolutions. Higher-resolution genomic analysis techniques might have yielded greater diagnostic rates. Despite its limitations, the study offers valuable insights into diagnostic yield among distinct patient subgroups, helping identify the patients who most likely to benefit from testing and enhancing our understanding of genomic copy number imbalances for future research.
